# Cancer

**Published:** 1915-07

**Authors:** 


### Cancer

Considered sociologically, the following facts seem pertinent: While local variations in incidence occur, no direct and invariable tendencies have been established as regards inherent natural factors of the subject (race), or of the environment (geologic formation, climate, altitude, water supply, etc.).

No clinical evidence of a parasitic, infectious nature, exists In other words, close association, attendance as nurse, physician, operator, post mortem examiner and other conditions which would almost inevitably lead to marked increase of incidence if an infectious element existed, even in the absence of frank contagiousness, does not in general predispose to cancer, although individual case histories suggest such a view. Cancer houses have been shown to be, in the main, those multiplying the chances of incidence by long occupancy, occupancy by a larger number of families or individuals than the average, occupancy by the elderly. Heredity has not been established by any careful comparative statistics.

The only scientific, experimental reasons for viewing cancer as an infection are: the more or less satisfactory demonstration in cancerous tissues of so numerous and widely different micro-organisms as to lead to scepticism from this reason alone and none of which has been generally accepted as the specific cause; the occurrence of epidemics in laboratory animals of conditions histologically resembling cancer but differing in many important respects, occasional accidental or intentional implantation of cancer, which does not imply the existence of an exogenic parasitic factor.

No mechanic or traumatic factor has been especially connected with cancer, except prolonged and continued irritation and certain lacerations mainly due to child-birth and affecting the uterus.

No chemic or metabolic factor has been especially connected with cancer, excepting only such as may be considered secondary to this or any analogous chronic disease or associated with senility, with the possible exception of the excess of cancer in parts subject to acid secretion or fermentation, pointed out by the editor some years ago. This association is, however, largely explained by histologic and physiologic coincidence and, at most, acts in analogy to prolonged mechanic insults.

The plausible theory that cancer was simply one phase of a general condition, limited by definition to certain tissues and occurring in its essential nature in other tissues (as sarcoma for instance) has not been established.

Attempts to connect cancer with diet, either on a parasitic or metabolic basis have failed, except that it is possible that, in a quantitative sense, further studies may establish a contributory etiologic relation for certain food stuffs.

While cancer is apparently increasing, it is probable that this is due to more careful reports, to a relative diminution of the younger element in the average population and to an increase of longevity, with diminution of other causes of death. While it is possible that a more scientific study of statistics may still demonstrate an increase in cancer incidence, such as is claimed from superficial inspection, it is highly improbable that the increase is important quantitatively or significant etiologically.

Sexual differences in the incidence of cancer are almost solely due to the presence in the female of additional potential histologic sites of development, subject to the factor of prolonged mechanic irritation. We can almost say, mathematically that the female cancer incidence equals that of the male, plus that due to the vulnerable and vulnerated tissues by which the female exceeds the male, and minus the incidence in non-sexual parts which, by the law of chance, would be spread over this excess of female parts.

The one positive fact which stands out from this mass of negative statements, is the unquestionable relation of cancer to senility, or rather to age. As the writer has pointed out, this relation is far greater than appears superficially. If we judge each age-group by itself, we find that 9.07% of all deaths of males between 60 and 64 are due to cancer and 18.06% of those of females between 50 and 54. But even this relation between cancer and age is not so direct as might appear. No age is exempt. While we may say that cancer is due to senility we cannot say that it is due to senile changes in any strictly histologic sense for such changes are not especially well-marked in the cancerous as compared with the non-cancerous nor are they present precociously in early cancer. Unless we assume a far longer latent stage of cancer than has been demonstrated on any large scale, we can not say that cancer is, so to speak, precipitated either by the climacteric in the frank sense or by any analogous conception of a definite beginning of the senile period. The epigram, which the writer has himself been guilty of repeating, that cancer develops in the adolescence of senility is untrue, unless a prolonged average period of latency can be established. Neither do the tendency to cancer and the degree of senility coincide. From the maxima of incidence stated above, the curve of frequency of cancer deaths as related to total deaths in any age-group, declines almost as rapidly as it has risen in earlier years.

It would appear from these facts, not that there is a genuine predisposition to cancer by any phenomenon associated with senility but that there is an average predisposition—not conspicuously nor even appreciably associated with any kind of exogenic or even extrinsic cause—slight for any one year, almost submerged by the high death rate from other causes in infancy and only occasionally appearing above the surface of the still enormously more frequent causes of death of other nature, during adolescence and early adult life; gradually become prominent as time is afforded for the operation of the law of chance and as other causes of death are avoided, survived or actually prevented by some sort of immunity; further increased by the operation of prolonged and repeated contributory causes, not individually of any specific etiologic importance; diminishing as extreme old age is reached, both by the increasing submergence in other causes of death as in early life and, perhaps, because of a more and more signal resistance to cancer itself.

Nothing can be more definitely established than that a cancer, once developing as such, tends to multiply both in its original site, in metastases, even in favorable condtions when transplanted to another organism. By analogy, we naturally think of some original extraneous stimulating cause, first of an essentially parasitic nature, either vegetable or animal, next—in the absence of a demonstrated germ—of some particular and limited mechanic, chemic or vibratory stimulus. Some such cause, more or less strictly specific, may ultimately be discovered but there is no absurdity in the conception that the cancer cell—a primordially or accidentally modified epithelial cell—is its own germ. At present, the negative results of the most diligent search for such a cause, as well as certain general facts given, rather tend to justify the conception of cancer as an entity. This is not the expression of an opinion but merely the weighing of evidence.

Such a view of cancer is unfortunate since, if correct, it eliminates prophylaxis in the proper sense of the word and limits even the practical conception of prophylaxis to the avoidance of any kind of prolonged stimulation of epithelial misgrowth, and to the prompt eradication of cancer in its incipiency.—which latter is an even less proper prophylaxis.

It should be clearly understood that the writer holds no brief for this conception of cancer and depends upon no personal experience or bias of a peculiar kind. General experience has simply been summarized and conclusions drawn in accordance. The statements made should be carefully scrutinized and, if incorrect in any particular, should be revised.

